# Photobiomodulation therapy and transcutaneous electrical nerve stimulation on chronic neck pain patients: Study protocol clinical trial (SPIRIT Compliant)

**DOI:** 10.1097/MD.0000000000019191

**Published:** 2020-02-21

**Authors:** Érika Patrícia Rampazo, Ana Laura Martins de Andrade, Viviane Ribeiro da Silva, Cláudio Gregório Nuernberg Back, Richard Eloin Liebano

**Affiliations:** Physioterapeutics Resources Laboratory, Department of Physical Therapy, Federal University of São Carlos (UFSCar), São Carlos/SP, Brazil.

**Keywords:** chronic pain, electrical stimulation, electrophysical agents, electrotherapy, lasertherapy, low-level laser therapy, neck pain, transcutaneous electrical nerve stimulation

## Abstract

**Introduction:**

Chronic neck pain is a common musculoskeletal disorder that is associated with functional disability and decreased of quality of life. Electrophysical agents are commonly used to relieve pain, however the effects of combined use of these agents are little studied. The objective is to investigate the efficacy of photobiomodulation and electrical stimulation to relieve pain, both in isolation and combined.

**Materials and methods:**

This a 4-arm randomized placebo-controlled trial with patient and evaluator blinded. This study will be performed in Department of Physical Therapy at Federal University of São Carlos, São Carlos/SP, Brazil. One hundred and forty-four patients with chronic neck pain will be randomized into 4 groups: active photobiomodulation therapy with active electrical stimulation, active photobiomodulation therapy, active electrical stimulation, or placebo treatment. They will receive 10 sessions of treatment. Primary outcome: pain intensity (measured by pain numerical rating scale) posttreatment. Secondary outcomes: pain during movement, neck disability, range of motion, pressure pain threshold, temporal summation, conditioned pain modulation, depressive symptoms, pain catastrophizing, quality of life, analgesic intake, and global perceived effect at posttreatment (10 sessions). Pain intensity and global perceived effect will also be measured after 6 weeks randomization.

**Discussion:**

The findings of this study might clarify the importance of using the photobiomodulation therapy and transcutaneous electrical nerve stimulation for patients with chronic neck pain.

**Trial registration:**

NCT04020861. https://clinicaltrials.gov/ct2/show/NCT04020861?term=NCT04020861&draw=2&rank=1.

## 1 Introduction

Chronic neck pain is defined as pain or discomfort in the posterior cervical region between the superior nuchal line and the first thoracic spinous process and/or shoulder girdle with or without pain referred to the upper back or arms and continuing for at least 3 months.^[1–3]^ Neck pain is considered non-specific when it is not related to any specific pathology such as inflammatory rheumatic disease, osteoporosis, cancer, or radiculopathy.^[2]^

Neck pain has an incidence of 10.4% to 21.3% and a prevalence of 17.1% to 73%^[1]^ and it is increasing with population aging.^[4,5]^ It is estimated that 71% of the population will experience neck pain some time in their lives with women being more likely than men to experience it.^[1,4]^ According to Global Burden of Disease, in 2015, more than a third of a billion people had neck pain of >3 months duration and neck pain was ranked the fourth leading cause of disability-adjusted life years (DALYs) globally just after ischemic heart disease, cerebrovascular disease, and lower respiratory infection.^[5,6]^ With such a large population affected by this problem, increasing knowledge about effective treatments for it should be considered a global health priority.

Photobiomodulation therapy (PBMT) and analgesic electrical currents are non-pharmacologic resources used in the treatment of patients with neck pain.^[4,7–9]^ PBMT is light therapy that uses lasers (light amplification by stimulated emission of radiation) or LED's (light emitting diodes) from the visible to the infrared spectrum, a portion of the spectrum where light interacts with chromophores leading to photophysical and photochemical reactions in tissues.^[10]^ Low-level laser therapy is nonthermal and it may have a stimulating effect on target tissues.^[11]^ Therefore, it is used in several musculoskeletal conditions to decrease pain and inflammation as well as stimulate collagen metabolism and wound healing.^[11]^ Côté et al,^[8]^ in their clinical practice guidelines, recommend the use of PBMT in combination with patient education for the treatment of chronic neck pain and associated disorders. This recommendation was based on 6 randomized clinical trials (RCTs)^[9,12–16]^ in which PBMT was better than placebo treatment and, in the majority of studies, the neck pain was associated with myofascial pain syndrome.^[8]^ Having been established as an effective treatment, it is then important to verify whether PBMT is superior to other analgesic agents including electrical currents.

Normally, electrical currents are applied through adhesive electrodes over the skin surface. This method is known as transcutaneous electrical nerve stimulation (TENS).^[17,18]^ Typically, TENS units deliver pulsed electrical currents with either balanced asymmetrical or symmetrical biphasic rectangular waveforms in which frequency, pulse duration, and amplitude can be adjusted.^[17,18]^ It is widely used in both acute and chronic painful conditions.^[17–19]^ Some studies have hinted that TENS might be more effective than placebo or as effective as other interventions for patients with neck pain.^[4,7,20–24]^ Unfortunately, the evidence is of low quality, the studies are heterogeneous, and more trials should be performed with larger patients samples, higher quality, and attention to adequate principles of application and evaluation of TENS.^[25]^

Both therapies, PBMT and TENS, are commonly used in clinical practice for patients with neck pain. The use of these electrophysical agents is important to decrease pain and may have the added benefits of decreasing the use of painkillers and facilitating exercise during therapy. However, the literature is still controversial, the studies are heterogeneous and of low quality and most of them only investigated pain and functional impairment. Therefore, more high-quality trials are required to verify the efficacy of these agents and the best methods of applying them. In addition, until this moment, no studies were found that investigated the combined effect of PMBT and TENS. These electrophysical agents have different ways of producing analgesia so we hypothesize that combined treatment with PMBT and TENS may have a synergistic action and result in a decrease in pain faster and/or for a longer time. Therefore, the objective of this study is to verify the efficacy of PBMT and TENS, isolated or combined, in relation to pain, functional disability, range of motion, pressure pain threshold, temporal summation, conditioned pain modulation, depressive symptoms, pain catastrophizing, quality of life, analgesic intake, and global perceived effect in patients with non-specific chronic neck pain.

## 2 Materials and methods

### 2.1 Ethical aspects and study design

**Figure 1:**
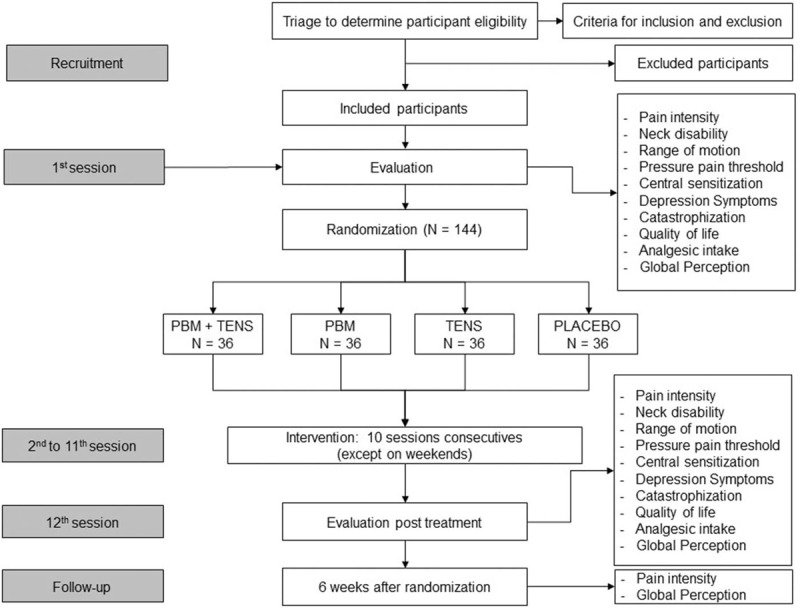
Flowchart of the study.

This protocol study was written following the recommendations of Standard Protocol Items: Recommendations for Interventions Trials (SPIRIT) and this 4-arm randomized placebo-controlled superiority trial with patient and evaluator blinded to the allocation group will follow the guidelines recommended by Consolidated Standards of Reporting Trials (CONSORT). Figure 1 provides a flowchart of the study.

The study has been approved by the Human Research Ethics Committee of the Federal University of São Carlos (UFSCar; CAAE: 81711417.0.0000.5504), São Paulo, on March 2018. The protocol of this study has been registered on Clinicaltrials.gov (NCT04020861). All the patients included in the study will validate their participation by signing an informed consent form that will be explained by evaluator.

### 2.2 Study setting

Patients will be recruited through the internet, posters, and radio dissemination. The study will be performed in Department of Physical Therapy of Federal University of São Carlos, Brazil, from January, 2020 until July 2019.

### 2.3 Eligibility criteria

The study evaluator will verify whether the patients will be eligible to participate in the study based on patient history and clinical examination.

### 2.4 Inclusion criteria

- Patients with non-specific chronic neck pain, defined as pain or discomfort in the posterior cervical region between the superior nuchal line and the first thoracic spinous process and/or shoulder girdle;
- Neck pain for at least 3 months;
- Neck disability index (NDI) score of 5 points or higher;
- Numeric rating scale (NRS) score of ≥3 for pain intensity;
- Aged between 18 and 65 years;
- Men and women.

### 2.5 Exclusion criteria

- Neck pain associated with nerve root compromise (measured by clinical examination of dermatomes, myotomes, and reflexes);
- Previous spinal surgery;
- Patients treated with physical therapy for neck pain within 3 months prior to the study;
- Severe spinal disorders such as fractures, tumors, inflammatory, and infectious diseases;
- Any contraindication to low-level laser therapy or transcutaneous electrical nerve stimulation;
- Rheumatic, metabolic, neurological, or cardiopulmonary diseases;
- Patients who require artificial cardiac pacemakers;
- Patients with sensory deficits;
- Skin diseases, mainly at the current application site;
- History of tumors or cancer in the last 5 years;
- Pregnancy;
- Having started any physical activity in the last 2 weeks.

### 2.6 Procedures

Patients will work with 2 separate researchers, 1 serving as the evaluator and the other serving as a therapist. Both are physiotherapists. The patients will be evaluated before the treatment, after the treatment (10 sessions) and 6 weeks after randomization. On the first session, the evaluator will collect sociodemographic data, medical history, and data related to the study outcomes. Then, the therapist will give each patient 10 consecutive days of treatment, with the exception of weekends, after which they will be re-evaluated by the evaluator. After 6 weeks randomization the evaluator will be in contact by telephone to the patients for a follow-up evaluation. All data entry will be coded and double-entered into an Excel spreadsheet for analysis.

### 2.7 Intervention

The patients will receive 10 consecutive days of treatment, with the exception of weekends. Each session will last around 1 hour, and it will be conducted by the same therapist during the same period of the day. After the initial evaluation, the patients will be randomly allocated to one of 4 groups: PBM + TENS group, PBM group, TENS group, and Placebo group. In the PBM + TENS group (n = 36) the patients will undergo the active PBMT and active TENS, in the PBM group (n = 36) the patients will be undergo the active PBMT and placebo TENS, in the TENS group (n = 36) the patients will undergo the placebo PBMT and active TENS, and in the Placebo group (n = 36) the patients will undergo the placebo PBMT and placebo TENS.

The equipment Antares (Indústria Brasileira de Equipamentos Médicos - IBRAMED, Amparo, São Paulo, Brazil) will be used for active and placebo photobiomodulation therapy (PBMT). Table 1 shows the parameters that will be used in the active PBMT.^[26]^ The patient will be prone. If laying prone is not possible, they will be seated. The treatment area will be defined as the painful area. A simulation of laser application will be performed for the placebo PBMT. The cluster probe will be positioned on the painful area for the same duration as active PBMT, the equipment will be turned on and set, but the trigger will be not activated, and no beam will be applied.

**Table 1:**
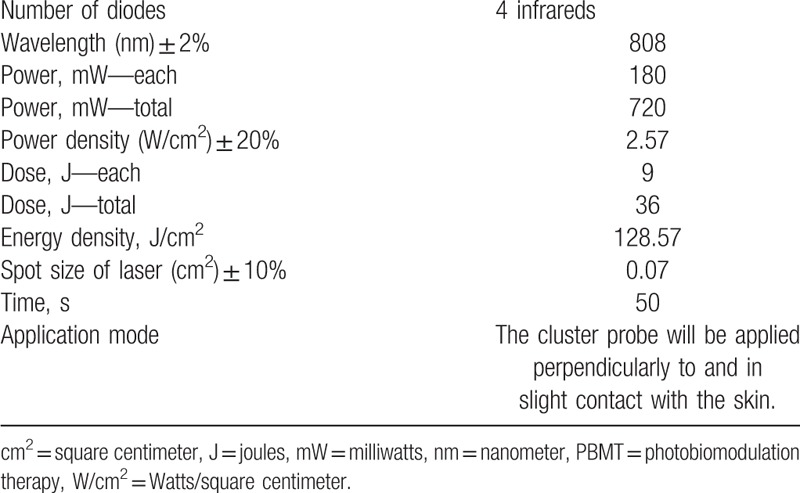
Parameters for PBMT.

The Neurodyn Portable TENS unit (Indústria Brasileira de Equipamentos Médicos - IBRAMED, Amparo, São Paulo, Brazil), which has a balanced asymmetric biphasic pulsed current, will be used for active transcutaneous electrical nerve stimulation (TENS). The patient will be prone. If laying prone is not possible, they will be seated. Two or four standard square self-adhesive electrodes (5 × 5 cm^2^) (ValuTrode, Axelgaard, CA) will be positioned around the painful area as reported by patient. The following parameters will be used: frequency of 100 Hz, phase duration of 125 μs, 30 minutes of current stimulation, and the pulse amplitude will be increased until the patient reports a strong but comfortable paresthesia (including motor level stimulation but no pain). The amplitude will be adjusted (if necessary) every 5 minutes to keep a strong but comfortable paresthesia. For placebo TENS, the device will be customized to deliver a current for 30 seconds (both channels) and then ramp off over the next 15 seconds so that it will be active only for a total of 45 seconds. This will permit the patient to feel the TENS sensation while applying the settings. The unit will also display an active indicator light suggesting to the patient that the unit is actively emitting current even after the 45 seconds. In a study comparing this placebo-TENS approach to standard placebo-TENS methods and active TENS, this transient placebo-TENS was found to improve blinding of evaluators without providing analgesia.^[27]^ Patients will be instructed to report when they feel the stimulation and every 5 minutes the patients will be asked if they are feeling comfortable.

### 2.8 Outcome measures

#### 2.8.1 Primary outcome

Pain intensity after treatment (10 sessions).

#### 2.8.2 Secondary outcomes

Pain intensity during movement; neck disability; cervical range of motion; pressure pain threshold (PPT); temporal summation (TS); conditioned pain modulation (CPM); depression symptoms; pain catastrophizing; quality of life; analgesic intake; and global perceived effect. Secondary outcomes will be measured at baseline and posttreatment (10 sessions). Pain intensity and global perceived effect will also be measured in contact by telephone after 6 weeks randomization.

### 2.9 Pain intensity—numeric rating scale

Pain intensity will be evaluated using a NRS, which is a simple and easy-to-use measuring scale that consists of a sequence of numbers from 0 to 10, in which 0 represents “no pain” and 10 represents “the worst pain imaginable.” Then, patients rate their pain based on these parameters.^[28]^ The pain evaluation will be carried out verbally with the patient reporting the pain intensity.

### 2.10 Neck disability—neck disability index

Neck disability will be evaluated using the neck disability index (NDI) that consists of a 10-item questionnaire that assess the impact of pain on daily activities using a score from 0 to 5 for each section, with higher values indicating more severe impact. This instrument has been translated and cross-culturally adapted for the Brazilian population.^[29]^ It will be used to include patients with neck pain in the study.

### 2.11 Cervical range of motion—fleximetry

Cervical range of motion will be measured with a fleximeter (Sanny, São Paulo, SP, Brazil). The intra-rater reliability of cervical range of motion was tested in 10 asymptomatic subjects by a single evaluator at 48-hour intervals. Reliability has already been estimated by calculating the intraclass correlation coefficients (ICC_2,3_) and it was reported as excellent for flexion (0.874; 95% CI: 0.473–0.969), extension (0.931; 95% CI: 0.716–0.983), inclination to the right (0.979; 95% CI: 0.919–0.995), inclination to the left (0.968; 95% CI: 0.873–0.992), rotation to the right (0.934; 95% CI: 0.746–0.984), and rotation to the left (0.832; 95% CI: 0.326–0.958).

To measure flexion and extension, the fleximeter will be positioned on the side of the head, over the ear, and the patients will be seated; for lateral inclination, the fleximeter will be positioned on the frontal region and the patients will be seated; for rotation, the fleximeter will be positioned at the central point of the head and the patients will be supine. Range of motion will be measured 3 times for each movement, and the mean value will be considered for statistical analysis.

### 2.12 Pressure pain threshold—algometry

Pressure pain threshold (PPT) will be measured using a Somedic Type II pressure algometer (Somedic, Hörby, Sweden) consisting of a circular rubber probe (1 cm^2^). The intra-rater reliability of measurement of PPT was tested in 10 asymptomatic subjects by a single evaluator at 48-hour intervals. Reliability has already been estimated by calculating the intraclass correlation coefficients (ICC_2,3_) and it was reported as excellent for the posterior cervical region and shoulder girdle (0.972; 95% CI: 0.887–0.993) as well as for the tibialis anterior muscle (0.985; 95% CI: 0.945–0.996).

For PPT measurement, the circular algometer probe will be positioned perpendicular to the skin and applied at a uniform and constant rate of 40 kPa/s. The patients will be instructed to close their eyes and to press the algometer sensor when the pressure sensation becomes painful. Three measurements will be collected with 30 seconds intervals between them and the mean will be used for data analysis. Patients will be not allowed to see the algometer readings during measuring. Six PPT recording points on cervical and shoulder girdle areas will be evaluated bilaterally: 2 cm lateral to the C2,^[30]^ C5,^[31]^ T4, and T8 spinous processes, at the middle point of the upper trapezius muscle (between C7 and the acromion)^[32]^ and the levator scapulae (2 cm superior to the superior angle of the scapulae)^[33]^ and on the middle third of the right tibialis anterior muscle.^[34]^ All patients will have 3 demonstrations of the PPT measurement on their right superior limb to ensure that they understand the PPT concept prior to starting the measurements.

### 2.13 Pain temporal summation

Temporal summation (TS) will be induced by a Somedic Type II pressure algometer (Somedic, Hörby, Sweden) with a circular rubber probe of 1 cm^2^. The intra-rater reliability of pain TS was tested in 10 asymptomatic subjects by a single evaluator at 48-hour intervals. Reliability has already been estimated by calculating the intraclass correlation coefficients (ICC_2,3_) and it was reported as good for upper trapezius muscle (0.710; 95% CI: 0.295–0.930). For TS, 10 stimuli will be performed with a pressure of 40 kPa/s up to the mean value obtained from algometry performed prior on the most painful upper trapezius or, if the pain is the same between the sides or there is not pain in the upper trapezius, the dominant upper trapezius. Each TS stimuli will be maintained for 1 second before being released and the stimuli will be spaced at 1 second intervals. A timer will be used to ensure that the intervals are respected and that the stimuli are maintained. Patients will be asked about their pain using NRS at the first, fifth, and tenth stimuli.^[35]^ To prevent sensitization interference from the previously performed pain pressure threshold evaluation, the test will begin 5 minutes after PPT evaluation.

### 2.14 Conditioned pain modulation

Conditioned pain modulation (CPM) represents a pain modulatory phenomenon in humans such that pain perception may be decreased by a nociceptive stimulus applied distantly from excitatory site.^[36]^ A noxious stimulus will be used as a conditioning stimulus to induce a reduction in the perception of pain from another test stimulus.^[37]^ The conditioned stimulus for eliciting CPM will be the cold pressor test and the test stimulus will be the assessment of PPT on the upper trapezius muscle.

First, the patients will receive clear instruction about the test procedure. Next, for the conditioned stimulus, a hand on the side ipsilateral to the most painful neck pain region will be immersed in a water bath maintained at room temperature (22 °C) for 1 minute to standardize the hand temperature.^[37]^ In the case of bilateral pain, the patient will be instructed to report the more painful side. If there is no consensus on which side is the most painful, the dominant side will be used. Thereafter, patients will be instructed to immerse the same hand (up to the wrist) in an ice water bath maintained at 4 °C.^[38]^ Patients will be asked to keep their hand moving (opening and closing the hand to prevent warming around the hand)^[39]^ in the water bath for 1 minute. During the conditioned stimulus, after 30 seconds, patients will rate the perceived pain intensity of the ice water on an 11-point verbal numeric pain rating scale with responses ranging from 0 (“no pain”) to 10 (“worst imaginable pain”). After 1 minute of immersion, the patient will be asked to remove their hand of ice water bath.

The PPT measure (test stimulus) will occur at the middle point of the upper trapezius muscle (between C7 and the acromion) contralateral to the immersed hand before the conditioned stimulus and, in order to avoid distraction bias, immediately after removing the hand from the ice water.^[40]^ For analysis of CPM efficacy, the mean PPT measured before the cold pressor test will be subtracted from the mean PPT measured after the cold pressor test. Hence, a lower CPM value reflects less efficient endogenous pain inhibition.

### 2.15 Depressive symptoms—the beck depression inventory

The depressive symptoms will be evaluated using the Portuguese version of the beck depression inventory (BDI). The scale consists of items including symptoms and attitudes whose intensity range from neutral to a maximum level of severity, rated from 0 to 3. It has 21 items related to sadness, pessimism, feeling of failure, lack of satisfaction, feeling guilty, self-deprecation, self-accusations, suicidal thoughts, crying crises, irritability, social retraction, indecision, distortion of body image, inhibition of work, sleep disturbance, fatigue, loss of appetite, weight loss, somatic concern, and decreased libido. Scores higher than 15 detect dysphoria and scores over 20 indicate depression.^[41]^

### 2.16 Pain catastrophizing—pain catastrophizing scale

Pain catastrophizing will be performed with the Portuguese version of the pain catastrophizing scale (PCS) validated and adapted by Sehn et al.^[42]^ The PCS is a self-administered questionnaire that consists of 13 items to assess catastrophizers. The items are rated on a 5-point Likert-type scale in which both intensity and frequency information are represented, with the following 5 levels of response for each Likert item: (0) not at all, (1) to a slight degree, (2) to a moderate degree, (3) to a great degree, (4) and all the time. The total score is computed by summation of all items and the total score ranges from 0 to 52 points. Higher scores indicate greater catastrophizing of pain.^[42,43]^

### 2.17 Quality of life—12-item short-form health survey—version 2

The quality of life assessment will be performed using the 12-Item Short-Form Health Survey (SF-12) version 2 questionnaire. This is a self-report measure that assesses physical (physical component summary—PCS) and mental (mental component summary—MCS) health on a scale of 0 to 100. Higher scores represent better levels of quality of life.^[44]^

### 2.18 Analgesic intake

All patients will be asked to report all analgesic medications (both opioids and non-opioids) taken 1 week prior to evaluation. The name, means of delivery, dose, pills per day, and number of days used in the past week will be recorded. All opioid medications will be converted into an equianalgesic dosage of oral morphine.^[45,46]^ Non-opioid analgesic medications will be converted to acetaminophen equivalents using the conversion table.^[47]^ During the treatment, the patient will be asked to record any medication used for their neck pain. These records will be important to determine if there will be a change in a medication dose per week used during treatment in relation to medication dose used week prior to the treatment. In addition, it will allow the researchers to determine whether the results will be biased as a result of medication use.

### 2.19 Global perceived effect—global perceived effect scale

The global perceived effect (GPE) scale, translated and validated for Portuguese, evaluates the patient's global perception of recovery. It consists of an 11-point scale that ranges from –5 (vastly worse) through 0 (no change) to 5 (completely recovered). At baseline, after the treatment, and at follow-up, the patients will be asked “Compared to when this episode first started, how would you describe your back these days?” A higher score represents a better condition.^[48]^

### 2.20 Randomization and blinding procedures

The randomization will be generated on the site www.randomization.com^[49]^ by a researcher not involved in the patient recruitment or data collection. It will be performed as block randomization with a 1:1:1:1 allocation. Patients will be stratified by sex to ensure equal numbers of women and men in each group and randomly allocated to 1 of 4 groups (n = 36 per group): Photobiomodulation + TENS, Photobiomodulation, TENS, or Placebo. The concealed allocation will be performed in consecutively numbered opaque envelopes. The envelopes will be sealed, and they will be stored in a secure cabinet. Prior to initiation of treatment, the therapist responsible for the treatment will open the sealed envelope to know in which group the patient will be included. Patient and evaluator will be blinded throughout the treatment. The same researcher will not apply therapies and perform evaluations.

### 2.21 Study blinding assessment

Assessment of the effectiveness of blinding will be performed after the conclusion of the posttreatment evaluation. The evaluator will answer whether he thinks that the application of photobiomodulation and electrical current was real, placebo, or he does not know. After that, the evaluator will ask the patient: “Do you think that the application of photobiomodulation was real, placebo, or did not know?” and “Do you think that the application of electrical current was real, placebo, or do you not know?” Their responses will be recorded and used to gauge the adequacy of subject and investigator blinding.

### 2.22 Sample size

The sample size of the study was performed based on the pain intensity outcome (as measured by the pain numerical rating scale) with mean difference of 2.3 points and an estimated standard deviation of 2.76 points.^[13]^ Statistical power of 80% was considered with an alpha of 5% and possible sample loss of up to 15%. Accordingly, a total of 144 patients will be required for the study. The sample calculation was performed using the Minitab software, version 17, (Minitab, Inc., PA).

### 2.23 Statistical analysis

All study-related information will be stored securely at the study site. All participant information will be stored in locked file cabinets in areas with limited access. The principles of intention-to-treat analysis will be used for the statistical analysis.^[50]^ The Shapiro-Wilk test will be used to verify the normality of the data. If the data present a normal distribution, a parametric test will be used. Otherwise, a nonparametric test will be used. The level of significance adopted will be *P* < .05. Data analysis will be performed using the SPSS software version 17 (SPSS, Inc., IL) by a researcher blinded to the division of the groups.

## 3 Discussion

This randomized controlled trial will investigate the effect of isolated and combined of photobiomodulation and electrical stimulation for chronic neck pain patients.

It will be possible to determine whether the application of one agent is superior to the other and/or whether the application of both is superior to isolated application of one of them, as well as whether they are superior to placebo treatment.

It will be possible to verify the efficacy of these agents not only in relation to intensity of pain but also in relation to range of motion, pressure pain threshold, central sensitization, functional disability, pain catastrophizing, depressive symptoms, and quality of life.

It has a high-quality design that leads to strong clinical evidence. The findings of this study might clarify the importance of using the PBMT and TENS for patients with chronic neck pain.

## Acknowledgments

“Coordenação de Aperfeiçoamento de Pessoal de Nível Superior – Brasil (CAPES) – Finance Code 001.”

## Author contributions

EPRS and REL were responsible for conceiving and designing the study. REL is the study coordinator. EPRS, ALMA, VRS, CGNB are responsible for data collection. All authors have contributed for writing and approved this manuscript.

Érika Patrícia Rampazo orcid: 0000-0002-2984-6902.
